# Supplementary material for: Antinociceptive, Sedative and Excitatory Effects of Intravenous Butorphanol Administered Alone or in Combination with Detomidine in Calves: A Prospective, Randomized, Blinded Cross-Over Study
Source: Animals (Basel). 2023 Jun 9;13(12):1943. doi: 10.3390/ani13121943 (PMC10295376; doi:10.3390/ani13121943)
Supplement: Supplementary file 1 [file animals-13-01943-s001.zip › animals-2356174-supplementary.pdf]

Supplementary Figure S1: For the evaluation of excitation, a scale based on behavioral items was created on purpose. The rating ranged from 0 (no excitation) to 3 (max excitation).

Universität Bern | Universität Zürich
vetsuisse-fakultät

SEDATION AND EXCITATION SCORES

VETERINÄR ANÄSTHESIE
UNIVERSITÄT BERN

Level	Sedation (Adapted from Ede et al. 2019)
0	No effect
1	Lowered head, braced stance, hindquarter weakness
2	Sternal or lateral recumbency, some responsiveness to enviroment
3	Sternal or lateral recumbency, no responsiveness to environment

Level	Excitation score
0	0 behaviours showed
1	1-2 behaviours showed
2	3-4 behaviours showed
3	≥ 5 behaviours showed

Excitation score

Pushing forward	Vocalization	________________
Jumping	Stepping	________________
Lifting the limbs	Urination	________________
Lateral flexion	Head tremors	________________

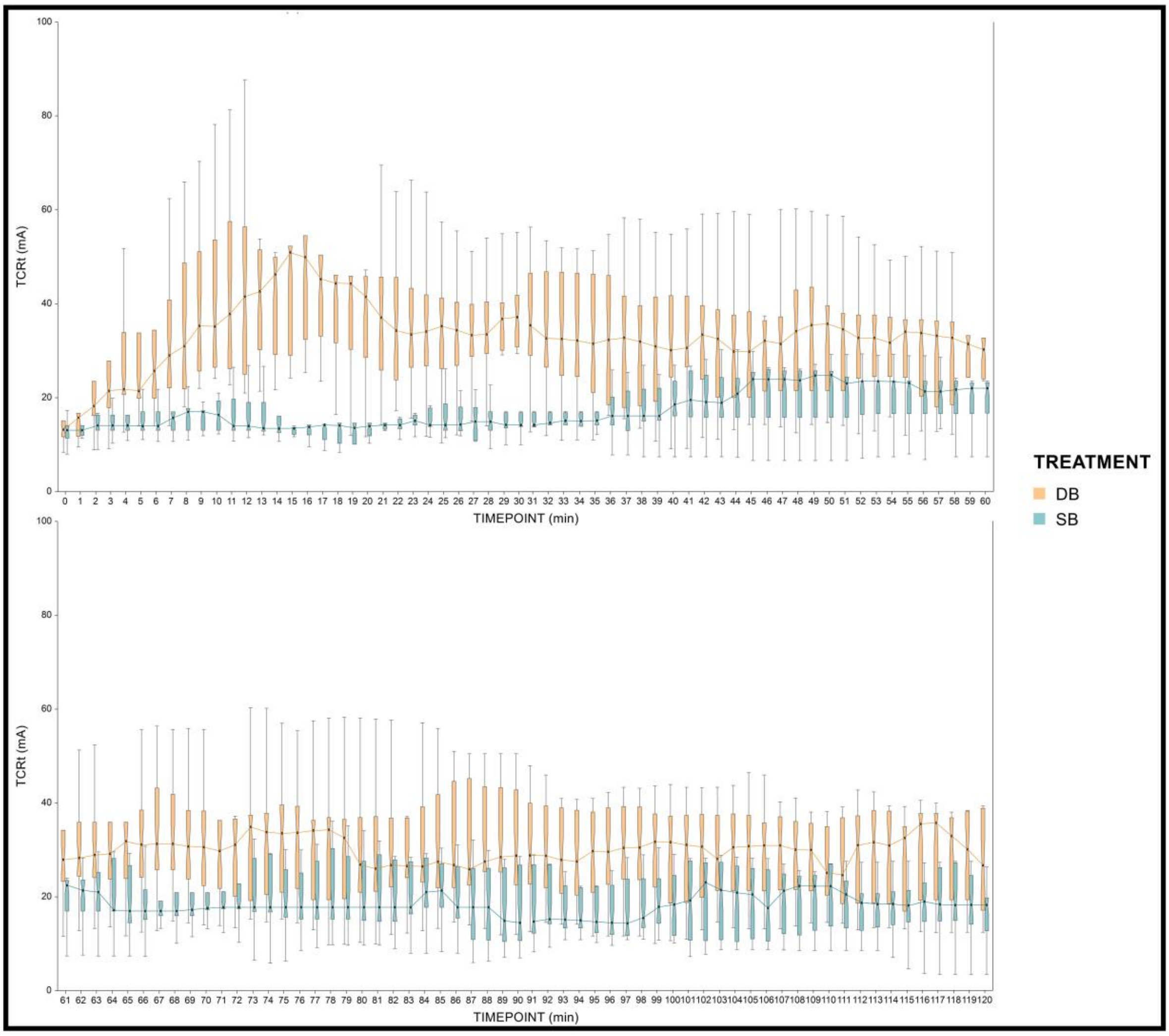

Supplementary Figure S2. Box-plot representing the median and interquartile ranges (25% and 75%) for the Trigemino-Cervical Reflex threshold over 1 minutes interval, from baseline up to 120 minutes. DB: Detomidine-Butorphanol (n = 8). SB: Saline-Butorphanol (n = 8). TCRt: Trigemino-Cervical Reflex threshold.
